# Mesoporous Silicon Microparticles Enhance MHC Class I Cross-Antigen Presentation by Human Dendritic Cells

**DOI:** 10.1155/2013/362163

**Published:** 2013-11-10

**Authors:** A. Jiménez-Periáñez, B. Abos Gracia, J. López Relaño, C. M. Diez-Rivero, P. A. Reche, E. Martínez-Naves, E. Matveyeva, M. Gómez del Moral

**Affiliations:** ^1^EM-Silicon Nano-Technologies SL, Nat. R. Cisternas 8, 46010 Valencia, Spain; ^2^Immunology Department, Faculty of Medicine, Complutense University, Avenida Complutense s/n, 28040 Madrid, Spain; ^3^Cell Biology Department, Faculty of Medicine, Complutense University, Avenida Complutense s/n, 28040 Madrid, Spain

## Abstract

The mesoporous silicon microparticles (MSMPs) are excellent vehicles for releasing molecules inside the cell. The aim of this work was to use MSMPs to deliver viral specific MHC class I restricted epitopes into human antigen presenting cells (monocyte derived dendritic cells, MDDCs) to facilitate their capture, processing, and presentation to CD8+ (cytotoxic) T lymphocytes. We show for the first time that MSMPs vehiculation of antigenic peptides enhances their MHC class I presentation by human MDDCs to CD8 T lymphocytes.

## 1. Introduction

Vaccines in general and virus vaccines in particular are focusing ever more on the induction of cellular immunity, specifically the generation of cytotoxic T lymphocytes (CTLs) [1–4]. Efficiency and safety issues arise with traditional vaccines, consisting of live attenuated or whole inactivated organism, so vaccine design nowadays focuses on the implementation of safer recombinant subunit vaccines [5]. These recombinant subunit antigens require potent adjuvants or immune modulators to enhance their immunogenicity as well as their capacity to trigger CTLs responses required to fend off life-threatening infections caused by intracellular pathogens, such as HIV, malaria, and tuberculosis [6]. The encapsulation of recombinant proteins in biocompatible and biodegradable nano- and microparticles is emerging as a promising approach to boost their immunogenicity by passively targeting them to antigen presenting cells (APCs) [7–9]. By mimicking pathogen dimensions, microparticles are more prone to be phagocyted by APCs than soluble antigen. The most powerful antigen presenting cells are dendritic cells (DCs), which bridge innate and adaptive immunity and are capable of initiating a primary immune response by activating naïve T cells [10]. The induction of most CD8+ T cell responses by DCs requires the presentation of peptides from internalized antigens by class I major histocompatibility complex (MHC) molecules that usually present endogenous cytoplasmic antigens. This process, essential for the efficacy of therapeutic vaccines, is called cross presentation, and DCs are the main antigen cross presenting and cross priming cell type *in vivo* [11].

In the last few years the biomedical research field has shown a growing interest in nanostructured silicon materials. Mesoporous silicon microparticles (MSMPs) possess unique chemical and structural properties such as chemical stability, adjustable pore size, extensive surface area, biocompatible and biodegradable nature, and notable cells adherence to its porous surface [12, 13]. These properties may offer large advantages over current adjuvants or vehicles in life science, namely, in drug delivery, tissue engineering, or gene therapy systems. Indeed, the use of mesoporous silicon materials has been investigated in a number of biomedical applications, including biosensing [14], tissue engineering and scaffolds [15], and, most recently, drug delivery [16–19]. In the present work we investigated the use of mesoporous silicon microparticles (MSMPs) for adjuvant and antigen deliver purposes. 

## 2. Materials and Methods

### 2.1. Mesoporous Silicon Particles (MSMPs) Preparation and Characterization

Due to novelty of mesoporous silicon material in biomedical research, a short introduction to its middle-scale fabrication is presented below with an essential chemical and structural characterization.

Mesoporous silicon material was fabricated by electrochemical treatment of the entire 4 inches silicon wafer in the 1 : 1 fluoric acid (48% HF) : ethanol (96% EtOH) electrolyte. The chemicals of analytical grade were purchased and used as received. Silicon wafers were from Si Materials, Germany, boron doped with a resistivity of 0.01-0.02 Ωcm (p+), wafer diameter of 100.0 ± 0.5 mm, and thickness of 525 ± 25 microns. Fluoric acid solution was from Riedel de Haën, Germany and ethanol from Panreac, Spain. Synthetic air (N_2_ with 21% of O_2_) was provided from AbelloLinde S.A., Spain and Milli Q water was used throughout the study.

The used electrochemical regime was as described: 40 mA/cm^2^ was applied for 5 seconds followed by 2.5 seconds of etchstop with zero current. This regime helps to achieve a uniform porous structure with homogeneous distribution of porosity and pore size across the deeply treated silicon wafer as well as to scale fabrication to few grams of material in one step. The periodic treatment was maintained during 3 hours until the practically entire wafer was converted into the porous material in a layer of approximately 350 nm thickness. The silicon substrate with a porous layer was then removed from the electrolyte, washed with distilled water, and dried in air. To stabilize the mesoporous material an additional thermal oxidation was performed under a synthetic air flow at 450°C during one hour (Ivoclar-Vivadent Technical Owen, Programat P200). 

**Figure 1:**
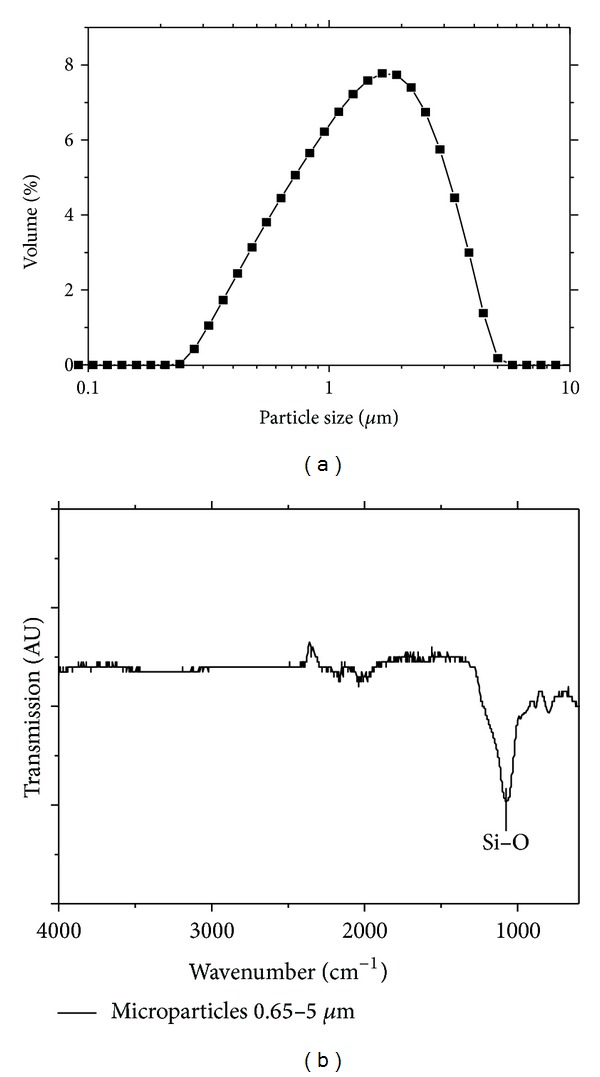
(a) Particle size distribution of MSMPs materials (nanosizer measurement) after being milled and sieved. (b) FTIR spectra of the porous silicon layers on a wafer after thermal oxidation process.

To obtain material with micrometer-sized particles, the mesoporous layer was mechanically removed from the wafer (approximately 2 grams of total weight), milled in air and sieved in cascade. For that the powder was suspended in distilled water and filtered through membranes with pore sizes of 5 and 0.66 microns successively. The fraction retained in between was used for further studies. To characterize the particle size distribution the laser backscattering optical method was employed (Nanosizer, Malvern Instruments, England), and in Figure 1(a) the measured distribution curve is shown thus confirming the accuracy of a preparation procedure—particles between 600 nm and 5 microns were found. 

Porosity of mesoporous material was determined gravimetrically by comparing the masses of the silicon wafer before and after electrochemical treatment, and the mass of the remnant wafer after removing the porous layer by scratching ([20], see Equation (1) in Supplementary Material available online at http://dx.doi.org/10.1155/2013/362163). To better adjust the last weight, the wafer was additionally washed in 1% KOH and then dried. The porosity calculated gravimetrically was as high as 71%.

**Figure 2:**
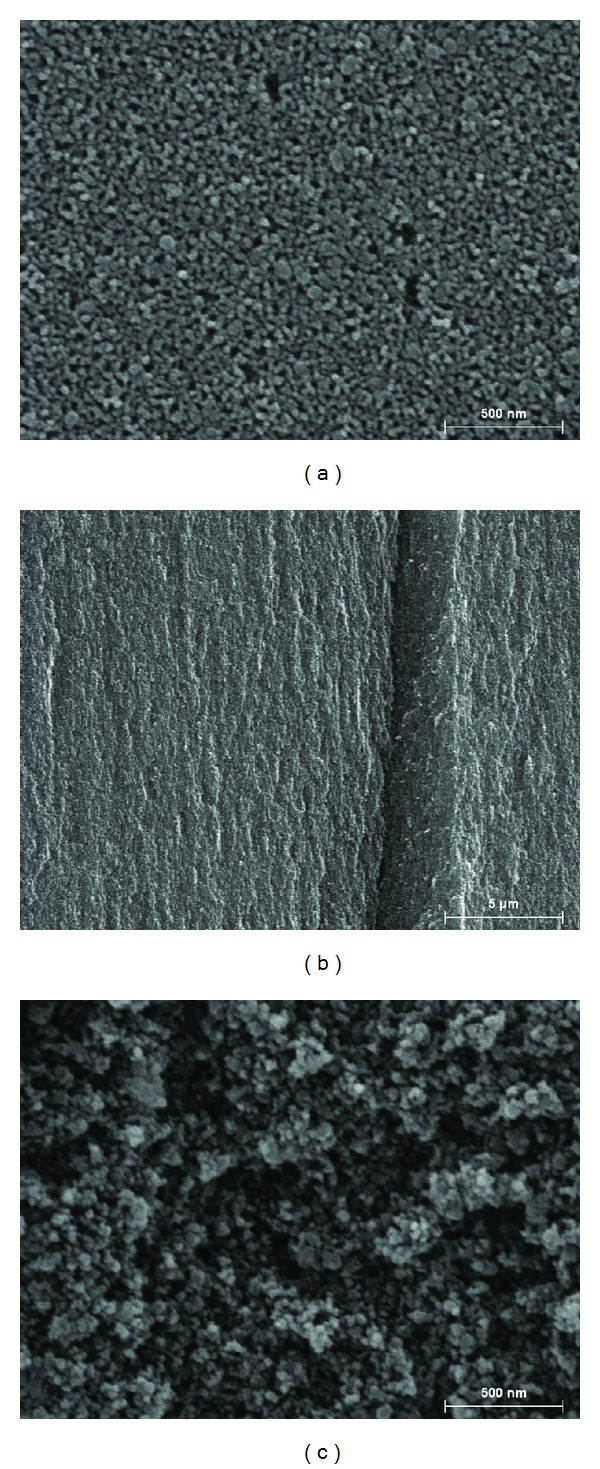
The scanning electron microscopy photos of the as-prepared porous silicon material: (a) in-plan view of the treated silicon wafer (×50.000), (b) transversal view of the porous material (×5.000), (c) particle surface after grinding (×50.000).

The surface area and pore size distribution were analyzed by nitrogen adsorption/desorption method (77.1 K, Micrometrics ASAP2000 volumetric analyser). Measurements were performed prior to the milling and after the milling and cells assays; samples were out-gassed under dynamic vacuum overnight at 130°C (for initial not milled material) or at only 37°C (after bioassays). Both the BET (Brunauer-Emmett-Teller) and the BJH (Barrett-Joyner-Halenda) approximations were used to calculate surface areas. The pore size distribution curves were calculated using the BJH method. Using the total volumes of adsorbed nitrogen and the weight of the porous sample used in each analysis, we were able to calculate the porosity as well. This method yielded values of 65–67%, quite similar to that obtained by gravimetric method. All the calculated data are presented in Table 1. The morphology of the initial material was visualized with the high resolution Scanning Electron Microscopy (SEM, Hitachi S4500). In Figure 2, the SEM images of the fabricated material are shown: (a) before scratching of the porous layer out from the Si wafer, (b) the transversal view of a big fragment of porous material and (c) the individual particle after milling. The pores of less than 20 nm penetrated throughout the material are clearly seen on these photos.

### 2.2. Preparation of Viral Peptide Coated MSMPs and *In Vitro* Release Study

We selected a panel of 23 peptides ranging from 8 to 11 amino acids consisting of viral-specific CD8 T cell epitopes from influenza virus (Flu), *Cytomegalovirus* (CMV), and Epstein-Barr virus (EBV) (CEF Peptide Pool; Mabtech, Sweden) [21]. In order to load the CEF peptide pool (CEFpp) we have used MSMPs, with size between 0.65 and 5 microns, a pore diameter centered on 10 nm and rusty surface. To load the microparticles, 10 *μ*g of the CEFpp was dissolved in 50 *μ*L of phosphate buffer (PBS; pH 7.4) (200 *μ*g/mL) with a fixed amount of MSMPs (2 mg; 10^7^ MSMPs) during 24 hours under shaking at 4°C. As an indirect estimation of particle loading, we performed peptide release measurements. To assess whether the CEFpp was released from MSMPS, CEFpp loaded MSMPs were spun down (10 min. 10000 g, 4°C), the supernatant was eliminated, and CEFpp-MSMPs were resuspended in 50 *μ*L of PBS and incubated 24 hours at room temperature. After centrifugation (10 min. 10000 g, 4°C), the CEFpp concentration was measured in a nanodrop spectrophotometer at 280 nm. The concentration of the CEFpp was always between 40 to 60 *μ*g/mL (2-3 *μ*g). These results reveal that at least 20–30% of the present peptides present in the original CEFpp solution were loaded in the MSMPs. Taking into account that 1 *μ*g/mL of peptides corresponds approximately with a concentration of 1 *μ*M we can estimate that, in average, every particle is capable of loading a minimum of 1.2 × 10^7^ viral peptides.

### 2.3. Generation of Human Monocyte Derived Dendritic Cells (MDDCs)

Monocytes were obtained from buffy coats from healthy blood donors by means of Ficoll gradient centrifugation and magnetic cell separation with anti-CD14—conjugated microbeads (Miltenyi Biotec). Since immature dendritic cells are highly phagocytic, we used them in the uptake experiments. In order to obtain immature dendritic cells, monocytes were resuspended into 24-well culture dishes at a density of 1 × 10^6^ cells/mL and cultured in RPMI-1640 (Invitrogen), 10% FCS (Harlan Indianapolis, IN) supplemented with 1000 U/mL of rhIL-4, and 1000 U/mL of rhGM-CSF (ImmunoTools, Friesoythe, Germany) for 5 days. MDDCs were treated for 48 hours with MSMPs (ratio 1 : 10) or CEFpp. MDDCs were also treated with 100 ng/mL of lipopolysaccharide LPS (Sigma-Aldrich, St. Louis, MO) for positive control of MDDC maturation. Two days later, the MDDCs were harvested and used for further assays.

### 2.4. Biocompatibility, Endocytosis, and Cell Surface Expression of DC Activation Markers

Cytotoxicity of MSMPs was assessed by incubating MDDCs generated as previously described with three different doses of microparticles (MDDCs/MSMPs: 1 : 10, 1 : 20, and 1 : 50) for 24 h, were collected and living cells were counted using Trypan blue dye under light microscope in a Neubauer chamber. All subsequent experiments were performed with a ratio MDDCs/MSMPs equal to or less than 1 : 20. 

The internalization of the MSMPs was assessed by flow cytometry with a FACSCalibur cytometer (Becton Dickinson, San Jose, CA) see Supplementary Material; Figure S2. Flow cytometry detected great differences in the complexity (SSC) of MDDCs exposed to microparticles compared to unexposed MDDCs, and SSC parameter was used to quantify the uptake of MSMPs. For that reason we did not label MSMPs with any fluorescent dye. The microparticle uptake imaging studies were performed with inverted and light microscopes: MDDCs exposed or not to MSMPs were collected at different times, spinning down on slides (500.000 cells/mL; 5 minutes; 300 g), and stained with hematoxylin/eosin. Three different fields at 200x were counted under light microscope. 

The cell surface expression of DC activation markers after stimulation of the MDDCs with medium, LPS, MSMPs or CEFpp was assessed by flow cytometry. Primary conjugated antibodies used in this study were as follows: anti-human CD86-fluorescein isothiocyanate (clone 2331 FUN-1), anti-CD80-PE (clone I307.4), anti-HLA-DR-PE (clone TU36l; BD PharMingen), anti-HLA-class I (A, B, and C; clone W6/32; American Type Culture Collection). For surface staining, cells were washed and incubated with specific antibodies for 30 minutes at 4°C. 

### 2.5. Endotoxin Quantitation on Silicon Microparticles (MSMPs)

MSMPs, CEFpp-loaded MSMPs, and free CEFpp were tested for endotoxin activity using a chromogenic LAL assay according to the manufacturer's protocol (LAL Chromogenic Endotoxin Quantitation Kit). LPS was included as positive control, and the sample (BLK) included in the kit was used as a negative control. 

### 2.6. Specific Antigen CTL Presentation Assay: IFN Gamma ELISPOT Assay

Antigen-specific CD8 T cells producing IFN gamma were measured by ELISPOT. The CEFpp (described above) consist of viral-specific MHC class I restricted T cell viral epitopes, and thus we attribute the production of IFN-*γ* to CD8+ T cells. The assay was essentially carried out in 96-well plates as described by Currier et al. [21]. Briefly, 100000 peripheral blood mononuclear cells (PBMCs) per well were incubated on IFN gamma moAb coated ELISPOT plates (Millipore, MA) with 20000 MDDCs pretreated with MSMPs loaded with CEF pool for 24 h at 37°C (Millipore, Temecula, CA). For each condition, the assay was run in triplicate. Positive controls were obtained by incubating PBMC with phytohemagglutinin (PHA). Negative controls were obtained by incubating PBMC with medium alone (negative control) and with MDDCs pretreated with peptide empty MSMPs. The number of specific IFN-*γ* secreting T cells was determined with an automated ELISPOT reader (Cellular Technology Limited, Germany), calculated by subtracting the average negative control value and expressed as the number of spot forming cells (SFC) per 10^6^ input cells. A response was considered positive if there were 50 SFC per 10^6^ input cells, and the activity was at least three times greater than the mean background activity.

### 2.7. Statistical Analysis

Mean values were compared by using the unpaired Student's *t*-test. All statistical analyses were performed with the Statgraphics program (Statpoint Technologies, Warrenton, VA). Statistically significant differences were represented as follows: *P < 0.05, **P < 0.03, and ***P < 0.01.

## 3. Results

### 3.1. MSMPs Are Efficiently Uptaken by MDDCs

**Figure 3:**
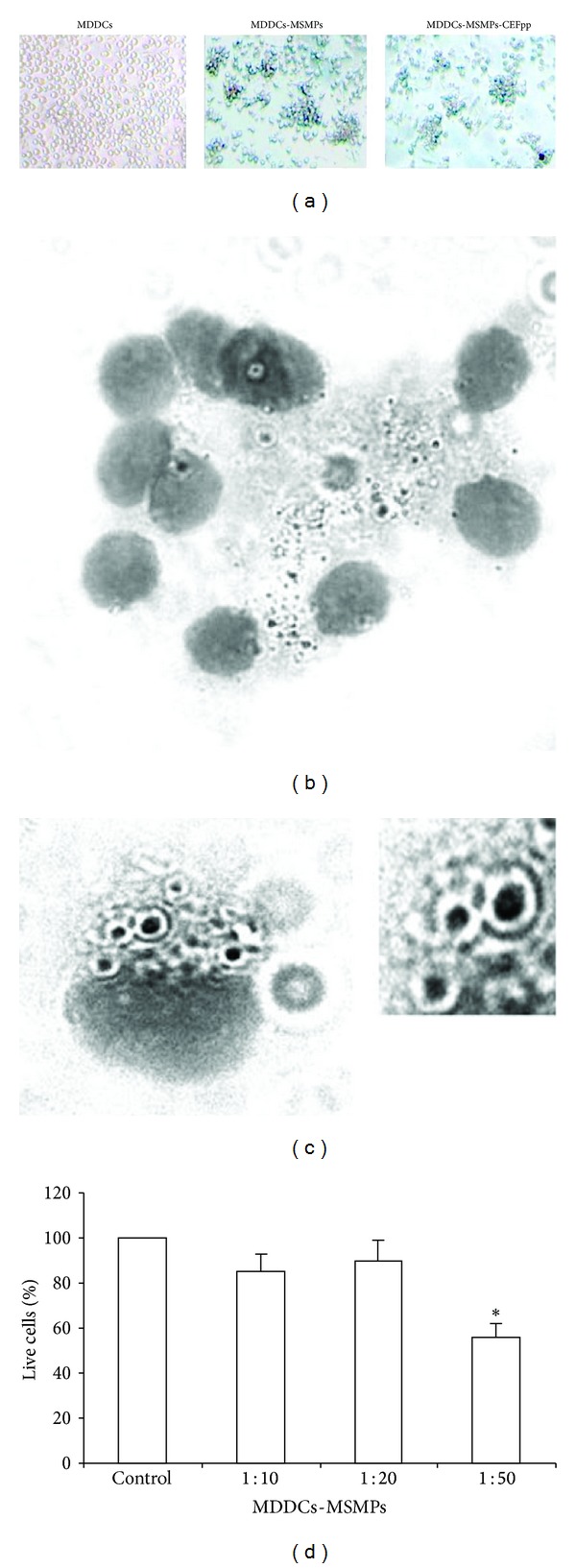
The microparticles are internalized into dendritic cells (DC). (a) Photographs show control cells (MDDCs) or with internalized unloaded (MDDCs-MSMPs) or peptide loaded (MDDCs-MSMPs-CEFpp) microparticles. After 24 h of incubation the cells are aggregated, and the particles are inside vacuoles ((b)-(c) and insert). Trypan blue dye was used to determine the percentage of live MDDCs relative to a nontreatment control following 24 h incubation with MSMPs (d). *P < 0.05.

After ensuring that MSMPs were not contaminated with endotoxin (see Figure S1 in Supplementary Material), initial experiments were performed to determine the best microparticle-MDDC incubation conditions to accurately measure and visualize intracellular microparticles. The best microparticle-MDDC ratio was 10 : 1. After 2 h approximately 30% of MDDCs contained embedded microparticles in membrane veils and endosomes. At 24 h large MDDC clusters with multiple engulfed microparticles in endosomes could be observed under light microscope. At this time 100% of human MDDCs had microparticles engulfed (Figures 3(a)–3(c)). Flow cytometry detected great differences in the complexity (SSC) of MDDCs exposed to microparticles compared to unexposed MDDCs, no differences were observed in size (FSC) between exposed or not MDDCs (see Figure S2 in Supplementary Material).

### 3.2. Toxicity of MSMPS in MDDCs

Cytotoxicity was assessed by incubating MDDCs with 3 different doses of microparticles for 24 h (Figure 3(d)). Cells treated with 10 or 20 microparticles per MDDC did not present any significant differences in a percentage of live cells compared to the control cells. At a highest dose of microparticles (50 per cell) there was a significant decrease in the percentage of live cells after incubation (60%; *P* < 0.05).

### 3.3. MSMPs Augment the Antigen Presentation Capacity of MDDCs

**Figure 4:**
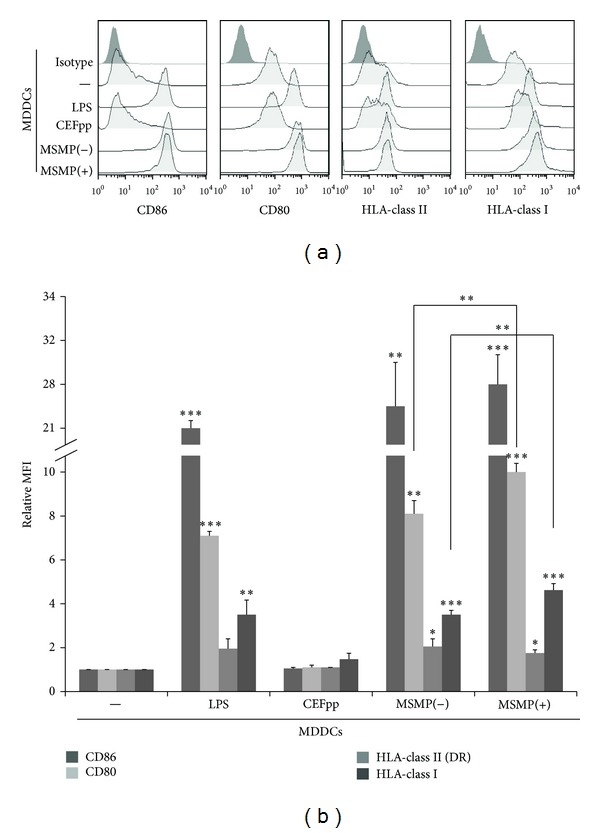
Dendritic cells maturation by MSMPs. (a) Representative histograms of dendritic cells maturation determined by quantifying the presence of the following activation markers: HLA-class I, CD80, CD86, and HLA-class II (DR) on their cell surface by flow cytometry. Isotype: irrelevant antibody; (−): MDDCs alone; CEFpp: MDDCs exposed to antigenic peptide (CEFpp) alone; MSMPs−: MDDCs with microparticles without CEFpp; MSMPs+: MDDCs with microparticles loaded with CEFpp; LPS: lipopolysaccharide. (b) Relative mean fluorescence intensity. Mean ± SD (*n* = 4). *P < 0.05; **P < 0.03; ***P < 0.01.

A hallmark of DC maturation is the upregulated expression of certain surface markers, namely, HLA and costimulatory molecules, involved in antigen presentation and stimulation of T cells. To elucidate how Silicon microparticles (MSMPs) affected the phenotypical maturation of human MDDCs, we analyzed cell surface expression of HLA-class I, HLA-class II, CD80, and CD86 (Figure 4). In order to standardize the values, we determined relative mean fluorescence intensity (MFI) by dividing the MFI of the treated population by that of the control untreated DC population. MSMPs induced a significant increase in HLA-class I, HLA-class II, CD80, and CD86 expression in MDDCs (*P* < 0.01). Expression of CD80, CD86 was slightly upregulated in all MSMPs treated DCs (with or without CEFpp) than in LPS treated MDDCs. Interestingly, HLA-class I expression was higher in MSMPS-CEFpp treated MDDCs as compared with MSMPs treated MDDCs (*P* < 0.01). On the contrary, human MDDCs treated with CEFpp alone showed only a nonsignificant small rise in both HLA-classes I/II compared with control immature MDDCs and no changes in CD80/CD86 (Figure 4(b)). These findings highlight that MSMPs are capable of activating MDDCs and indicate that MSMPs uptake influences MDDC phenotype and their ability to mature.

### 3.4. Enhanced Antigen Presentation by Human MDDC Loaded with MSMPs Containing Class I Restricted Peptides

**Figure 5:**
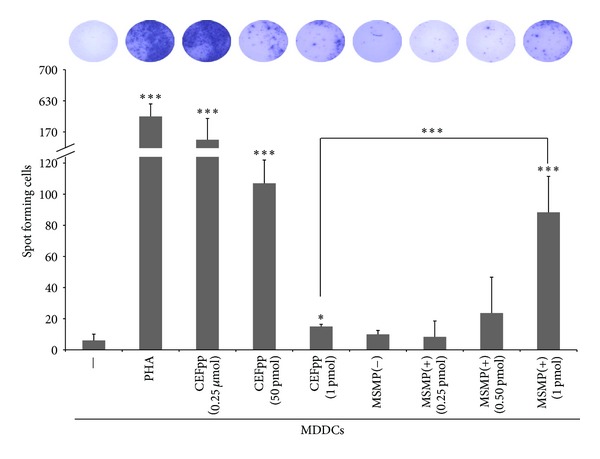
Porous silicon microparticles loaded with antigens trigger the immune response. ELISPOT assays: the incubation of peripheral lymphocytes (PBMCs) from healthy donors with allogenic dendritic cells (MDDC) differentiated and matured in the presence of microparticles loaded with antigenic peptides (MSMP+) results in the activation of specific CD8 lymphocytes, showed as a synthesis of IFN-*γ*. PHA: phytohemagglutinin (1 *μ*g); CEFpp: CEF peptide pool; MSMP(−): unloaded microparticles. *P < 0.05; ***P < 0.01.

To address the question whether MSMPs can enhance the activation of viral specific CTLs, we performed *in vitro* co-cultures of peripheral blood mononuclear cells (PBMCs) and MDDCs treated with MSMPs loaded with common viral specific CD8 T cell epitopes. Specifically, we determined whether our MSMPs microparticles loaded with the CEFpp could induce specific CTL responses as measured by IFN-*γ*-ELISPOT assays (see details in Material and Methods). As seen in Figure 5, CEFpp-loaded MSMPs clearly enhanced the T cell stimulatory capacity of MDDCs in comparison with CEFpp alone. Nonstimulated (PBMC alone) and nonloaded MSMPs were unable to stimulate T cells. In addition, the adsorption of CEFpp in silicon microparticles significantly promoted the antigen presentation capacity of MDDCs to human CD8 T cells at different ratios in comparison with immature and free CEFpp-stimulated MDDCs, clearly indicating the antigen dose-sparing effect of microparticle adsorption (Figure 5). A 50 fold higher initial concentration of free CEFpp to obtain similar IFN gamma SFC when compared with CEFpp-MSMPs (Figure 5) that could be explained by the fact that antigen presentation is enhanced in MDDC treated with MSMPs.

## 4. Discussion

MSMPs used in this study (mesoporous silicon materials, prepared by an electrochemical method [19] and dispersed in the form of microparticles) are able to stimulate an *in vitro* maturation of human dendritic cells after particles uptake and the following enhancement of specific viral peptide CTL response. Cytotoxic CD8+ T lymphocyte (CTLs) responses are critical for immunity against viruses and tumors [1–4] and the results shown here with mesoporous silicon microparticles make them attractive candidates for stimulating cellular immune response.

Considering the outstanding specific surface of MSMPs (~250 m^2^/g), we have chosen the adsorption method for loading virus peptides in these nanostructured carriers as was previously described by Pastor et al., 2011, to load insulin and albumin in the same microparticles. Adsorption of biopharmaceuticals from aqueous solutions is an ideal method for drug loading since it does not require high mechanical energy, use of organic solvents, or high temperatures, factors that might lead to the denaturation or the chemical degradation of protein drugs [19]. It is very likely that a large fraction of the peptides were loaded in an inner space of the carrier, since the radius of gyration of many peptides is well below the pore size of the prepared MSMPs (10 nm). We showed a load-release capacity of MSMPs between 20 and 30% of the original viral CEFpp content in a solution, enough to induce a strong immune response. We have to take into account that total loading capacity was limited by the MSMPs toxicity at high dose. According to previous reports, loading efficiencies can vary between 9% and 45% for similar particles at pH 7.4 [16, 19]. Adsorption process in MSMPs is a complex process, and in a future work we will focus on studying the possibility to control the protein adsorption parameters through surface modification or modulation of the carrier porosity to improve load-release capacity of MSMPs. We measure the MSMPs release capacity at only 24 hours. Studies on the insulin release kinetics from the MSMPs indicated that this process is very fast (>80% of insulin is released at 45 min) but controlled (burst release below 20%) [19]. Toxicity of MSMPs on MDDCs was very low, and it was previously demonstrated that other silicon microparticles are biocompatible with respect to endothelial and macrophage cell viability, morphology, mitosis, and cell cycle [13, 22, 23].

The development of vaccines engaging the adaptive cellular immunity requires carrier systems that are capable of delivering the vaccines agents onto the Antigen Presenting Cells (APCs), in order to facilitate a potent and prolonged antigen presentation [24]. The MSMPs uptake by human immature DCs is very efficient, and it has been well documented as porous Silicon microparticles are capable of being internalized in epithelial and phagocytic cells by endocytosis processes as well as pinocytosis and macropinocytosis, being shown to be excellent vehicles for the liberation of molecules or drugs within the cell [13, 18]. We could have skipped the MDDCs isolation step and exposed loaded microparticles peptides directly to PBMCs, but the response would be much lower since the uptake of the microparticles is not optimized in that scenario and taking into account that our MSMPs formulations have an effect on the maturation of human MDDCs. A similar result has been reported using polystyrene PLGA nanospheres, which induced the upregulation of the maturation markers HLA-DR and CD86 in both cord blood derived DC [25] and murine bone marrow derived DC [26]. In contrast, another study did not show any maturation effect of similar silicon microparticles on murine bone marrow-derived dendritic cells [27]. These discrepancies are most likely due to the differences in the cells, culture conditions, and particle preparations used in those studies. Enhancing the expression of costimulatory molecules enables DCs to better present antigens to T cells. Indeed, this phenotypical DC activation usually correlates with a functional DC maturation as evidenced by the activation and proliferation of naive T cells [28]. 

For the presentation of the viral specific CD8 T cell epitopes loaded onto MSMPs-CEFpp, the involved antigen presenting cells must be able to “cross present” the exogenous peptides onto MHC class I molecules by either the classical proteasome and TAP-dependent pathway or by an alternative TAP-independent pathway of antigen presentation [11]. Cross presentation of soluble proteins by DC can also occur, but it is extremely inefficient, as it usually requires the incubation with protein antigens at high concentrations. Remarkably, in this study the amount of peptides adsorbed in our MSMPs was significantly lower than the amount of peptides used to pulse the DC externally. Taking into account that only 20–30% of initial CEFpp (Figure 5 data) is loaded in MSMPs, a high efficiency in CTL activation can be associated with MSMPs rather than with free CEFpp, thus suggesting an intracellular cross presenting mechanism, instead of an external peptide change in free CEFpp. We suggest that this enhancement of antigen presentation is probably due to the slow hydrolysis of the MSMPs in the endosomes of the DC, which provides a continuous supply of peptide ligands for newly synthesized MHC class I and II molecules. Viral peptide adsorbed in mesoporous silicon particles would be protected from degradation (silicon microparticles are long-term stable at low pH (<6) [19]) and released slowly into the dendritic cells antigen processing pathways. This would allow for an increased duration of antigen presentation compared to free peptide that would be quickly degraded.

Functional maturation and enhanced presentation of CEFpp delivered to MDDC via MSMPs are reflected in the increased production of IFN-*γ* by CD8 T lymphocytes against the CEFpp. This viral peptide pool stimulates IFN-*γ* release from CD8+ T cells in individuals with defined HLA types (11 class I HLA-A and HLA-B alleles whose cumulative frequency is representative in >90% of Caucasian individuals) and is used in vaccine trials in ELISPOT assays [21].

In summary, the experimental evidence presented in this work confirms the excellent properties of MSMPs as devices capable of loading therapeutic peptides and promotes maturation of human dendritic cells that trigger a specific CTL response.

## 5. Conclusion

Mesoporous Silicon Microparticles (MSMPs) appear to be a new promising composite device for adjuvant and antigen deliver purpose in vaccine design. Here we have demonstrated for the first time, efficient silicon microparticle-facilitated loading of viral specific class I-restricted T cell epitopes to human MDDC. We have observed that the enhanced viral peptide presentation correlated with a more efficient generation of antiviral CTL response. The therapeutic potency of MSMPs based vaccines should be tested in prime-boost vaccinations to stimulate “*in vivo*” similar CD8+ T-cell responses and to improve effective prophylactic protection against virus challenge.

## Supplementary Material

Equation to calculate porosity of MSMPs; Endotoxin test and a representative example of flow cytometry parameters to measure MSMPs uptake by MDDCs are included in supplementary material.

## Figures and Tables

**Table 1:** Structural and surface parameters of the mesoporous samples (adsorption data).

Sample	BET area, m^2^/g	BJH area, m^2^/g	Mean pore size, absolute, nm	Preferred pore size interval, nm	BJH porosity (vol.), %	Gravimetric porosity, %
Initial, nonloaded, before milling, 130°C dry	223	267	8.6	5–15	65	71
After cells assay, nonloaded, 37°C dry	54	60	16	10–30	35	
After cells assay, loaded, 37°C dry	51	40	23	9–30	36	
