# Supplementary material for: Mesoporous Silicon Microparticles Enhance MHC Class I Cross-Antigen Presentation by Human Dendritic Cells
Source: Clin Dev Immunol. 2013 Nov 10;2013:362163. doi: 10.1155/2013/362163 (PMC3842076; doi:10.1155/2013/362163)

**Supplementary Material; Eq. 1**

**- Porosity**

Porosity of the used porous silicon material was determined gravimetrically using the equation:

$$P(\%) = \frac{m_1 - m_3}{m_1 - m_2} \qquad (1)$$

Where $m_1$ is the mass of the initial Si wafer used for anodizing (porosification), $m_2$ is the mass of the Si wafer after anodizing and $m_3$ is the mass of the remnant wafer after removing the porous layer by scratching and cleaning in 1M KOH solution. Porous material is completely eliminated from the wafer in these conditions.

**Supplementary Material; Figure S1**

- **MS-MPs are free of endotoxin.**

Mesoporous Silicon microparticles ($1x10^7$) and CEF pp pool (0,5 µg/ml) were tested for endotoxin activity using chromogenic LAL assay. LPS (1µg/ml) was included as a positive control and a sample blank included in the kit as a negative control. ***P < .01.

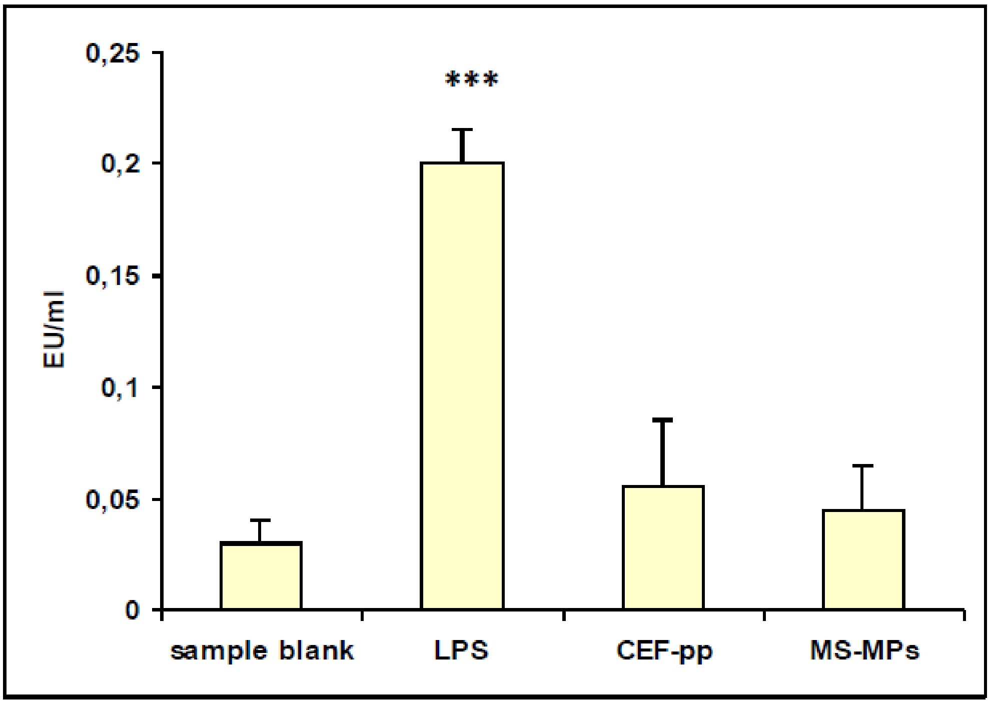

**Supplementary Material; Figure S2**

**Internalization of MSMPs by flow cytometry.**

A representative example of Forward (FSC) and Size (SSC) flow cytometry parameters to measure microparticle uptake by MDDCs at 24 h. Histograms (up) and mean counts (down) (D).

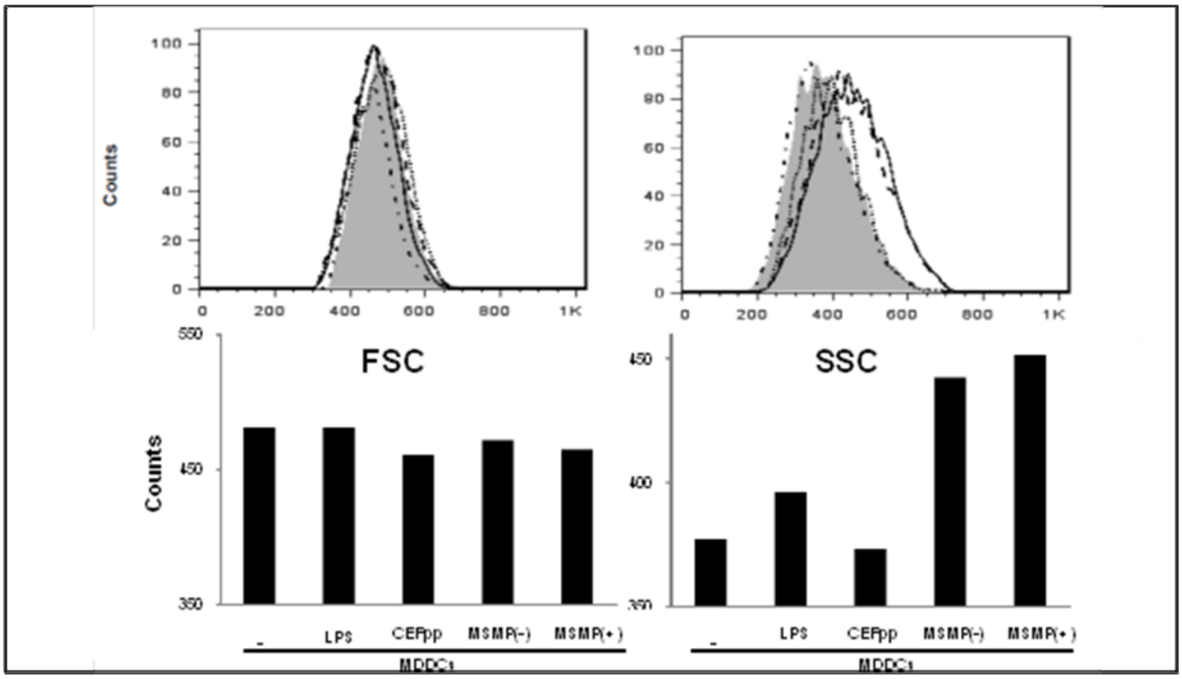

Supplement: Supplementary file 1 — Equation to calculate porosity of MSMPs; Endotoxin test and a representative example of flow cytometry parameters to measure MSMPs uptake by MDDCs are included in supplementary material. [file 362163.f1.pdf]
